# External financial dependence and firms’ crisis performance across Europe

**DOI:** 10.1007/s00181-021-02025-3

**Published:** 2021-02-26

**Authors:** Peter S. Eppinger, Katja Neugebauer

**Affiliations:** 1 University of Tübingen, Nauklerstr. 50, 72074 Tübingen, Germany; 2 Banco de Portugal, Lisbon, Portugal; 3 Systemic Risk Centre (London School of Economics), London, UK

**Keywords:** External financial dependence, Financial constraints, Financial crisis, Firm performance, G10, G30, L25, F14, G01, D22

## Abstract

How do financial market conditions affect real economic performance? Empirical investigations of this question have often relied on measures of external financial dependence (EFD) that are constructed using US data and applied to other countries under the assumption of a stable industry ranking across countries. This paper exploits unique, comparable survey data from seven European countries to show that correlations of EFD across countries are weak, casting some doubt on this assumption. We then use the novel survey-based EFD index to show that the global financial crisis had a disproportionately negative impact on the real performance of financially dependent firms. Further investigations highlight the importance of supply chains in propagating the credit shock.

## Introduction

How do financial market conditions impact on real economic performance? This question has been examined at least since Schumpeter (1911) and regained particular relevance after the global financial crisis that started in 2008 (see King and Levine 1993; Beck et al. 2000; Levine 2005; Beck 2012). (Rajan and Zingales 1998; henceforth, RZ) achieved significant progress towards establishing a causal effect of financial development on real growth by exploiting differences in external financial dependence (EFD) across industries. In their seminal contribution, RZ measure industry-level EFD as the share of investment not financed by internal cash flow in the median listed US firm (from the Compustat database) over the 1980s. Their approach rests on two main assumptions: First, if the US capital market is close to perfect, credit demand by listed US firms should not be contaminated by supply-side imperfections, but instead reflect technological fundamentals.^1^ Second, in applying the EFD index of US industries to other countries, RZ assume that the industry ranking is constant across countries. It is the second assumption that we investigate more closely in this paper.

Since the seminal contribution by RZ, their EFD index and updated versions of it have been used in many applications to different research questions and countries.^2^ For instance, Manganelli and Popov (2013) document nonlinearities in the relationship between financial development and growth, Dell’Ariccia et al. (2008) as well as Kroszner et al. (2007) examine how the real effects of banking crises vary by EFD, Manova (2013) uses the index to identify the role of credit constraints for international trade, and Chor and Manova (2012) analyze the differential impact of the global financial crisis on exports.

In this paper, we exploit a unique survey question in the EFIGE dataset^3^ to obtain a novel, country-industry-specific index of EFD for seven European countries. We first correlate industry rankings of EFD between this EFIGE index and an updated version of the Compustat index, computed from US data following RZ. To shed some light on the stable-ranking assumption, we proceed by examining correlations of the EFIGE index across countries.^4^ Both the Compustat index and the EFIGE index are then employed in firm-level regression analysis to examine the differential effect of the global financial crisis on the real performance of manufacturing firms across industries with varying EFD. Finally, we extend the regression approach to investigate the transmission of the credit shock in the crisis over time and across firms.

We find that industry rankings of EFD are weakly correlated across the two measures and also across European countries, which casts some doubt on the stable-ranking assumption. The subsequent regression analysis reveals that the crisis had a disproportionately negative impact on firm performance in financially dependent industries according to the EFIGE index of EFD, as can be expected. By contrast, the Compustat index delivers insignificant or counter-intuitive estimates. These findings point to the relevance of cross-country differences in industry rankings of EFD.

Our investigations into shock transmission, using the EFIGE index of EFD, provide only weak evidence for delayed differential effects of the financial crisis after 2010. More interestingly, we find significant propagation of the credit shock across firms along supply chains. Our regressions reveal that firm performance in the crisis was negatively associated with the EFD of a firm’s customers and, even more strongly, with the EFD of its suppliers. The estimates suggest that such credit-induced supply shocks played a sizeable role for firms’ crisis performance.

The paper is structured as follows. In Sect. 2, we characterize the data used and introduce the novel index of EFD based on the EFIGE survey. Section 3 shows how this measure correlates with the updated Compustat index and across countries. Section 4 exploits both EFD measures to examine how EFD affected firms’ performance in the financial crisis. The last section draws conclusions for future research.

## Data

To obtain the EFIGE index of EFD, we exploit the following question in the survey:

In the industry your firm works, how dependant [sic] are companies on external financing? To give your answer, please use a score from 1 (not dependent [at] all) to 5 (Extremely dependent).

Our novel index of EFD is the arithmetic mean of firms’ responses to this question by industry *j* and country *c*. This measure has three key advantages: First, it is directly comparable across seven European countries, as the identical question was posed to 14,364 (representatively sampled) manufacturing firms at the same time in 2010.^5^ Second, it mitigates reporting bias by addressing general conditions in the firm’s industry rather than the firm’s own financial situation. Third, its general formulation is designed to cover all relevant aspects of EFD.

Since the original Compustat index by RZ is not available for the European industry classification used in EFIGE (NACE Rev. 1.1), we follow RZ in computing the index from Compustat data on US firms. Each Compustat firm is assigned the NACE code corresponding to its SIC code. We select data from the more recent, pre-crisis period 1990–2005 and apply the measure to all countries for our subsequentA analysis, following the RZ assumption that EFD as a fundamental industry characteristic should be stable across countries and over time.^6^

For the analysis of firm performance, we merge the industry-level EFD measures to the Orbis firm dataset provided by Bureau van Dijk (BvD). Our panel includes 212,540 manufacturing firms from the seven EU countries under study over the period 2005–2013. It covers only firms (i) whose core activity is classified as manufacturing by their NACE code and (ii) which belong to the size classes medium, large, and very large, as defined by BvD. We compute real growth rates of performance variables (see Sect. 4), using producer price indices at the most disaggregate industry level that is available from Eurostat for each country (typically 4-digit NACE).

## Comparing industry rankings of EFD

Industry rankings of EFD for the updated Compustat index, computed from US data following RZ, and the survey-based EFIGE index by country are listed in Table 6 in “Appendix A”. Table 1 shows Spearman rank correlation coefficients for all pairwise comparisons between these rankings.

Two observations stand out. First, the ranking of US industries based on the Compustat index is not positively correlated with the rankings of EFD as perceived by European firms and reported in the EFIGE survey. Instead, the correlation coefficients reported in the first row of Table 1 are even negative for most countries except Italy and Spain, for which they are small and insignificant.

Second, when comparing the EFIGE index across countries, the industry ranking is highly unstable. Only for six out of 21 pairwise comparisons does the correlation coefficient exceed 0.3, and it is only significant at the 5% level (based on a two-sided *t* test) in three of these cases. The correlation is close to zero for most country pairs and even negative in eight cases.^7^

**Table 1:** Correlations of EFD rankings across countries

	AUT	DEU	ESP	FRA	GBR	HUN	ITA
USA (Compustat)	$-$ 0.2707	$-$ 0.0200	0.0889	$-$ 0.1680	$-$ 0.2087	$-$ 0.0652	0.1104
AUT (EFIGE)		$$-$$ 0.0767	**0**.**5609**$$^{**}$$	0.2887	$$-$$ 0.3699	$$-$$ 0.3263	$$-$$ 0.5414$$^{**}$$
DEU (EFIGE)			0.2739	**0**.**4279**$$^{**}$$	$$-$$ 0.1174	0.2925	**0**.**3600**$$^{*}$$
ESP (EFIGE)				**0**.**5178**$$^{**}$$	$$-$$ 0.1196	0.2105	$$-$$ 0.1937
FRA (EFIGE)					0.0761	$-$ 0.0446	0.0247
GBR (EFIGE)						0.2826	**0**.**3391**
HUN (EFIGE)							**0**.**3982**$^{*}$

The table shows Spearman rank correlation coefficients for pairwise comparisons between the rankings of EFD across countries listed in Table 6. The EFD index for US firms is computed from Compustat for 1990–2005, following RZ. The remaining measures are based on average values of reported EFD by industry and country from the EFIGE survey. Correlation coefficients exceeding 0.3 are marked in bold. Asterisks indicate significance levels based on a two-sided *t* test: *$p<0.10$; **$p<0.05$

Provided that the EFD score reported by firms for their industry is systematically related to the fundamental EFD, these observations indicate that (i) the Compustat index based on US data is uncorrelated with EFD in European industries and (ii) even within Europe, there are substantial differences in the industry rankings of EFD across countries. These findings cast some doubt on the stable-ranking assumption and suggest that it might be advisable to take country-specific factors into account when investigating the role of EFD.

## Firm performance in the global financial crisis

### Main econometric specification

We now use the Orbis panel dataset to analyze the differential impact of the global financial crisis on firms’ real performance depending on EFD. This exercise fulfills the double purpose of (i) assessing the detrimental impact of the crisis on firm performance through the credit channel, and (ii) evaluating the usefulness of the alternative EFD measures for this purpose.^8^

We estimate the following econometric model:

1 $$\begin{aligned} \Delta \ln Y_{it} = \beta \,{\hbox {Crisis}}_{ct} \times {\hbox {EFD}}_{cj} + \delta _{ct} + \delta _{i} + \varepsilon _{it}, \end{aligned}$$

where $\Delta \ln Y_{it}\equiv \ln Y_{it} - \ln Y_{i(t-1)}$ measures real growth in the performance of firm *i*, which is active in country *c* and industry *j*, in year *t*.^9^ We examine the following dimensions of firm performance $Y_{it}$: real turnover (operating revenues), employment (number of workers), real labor productivity (value added per worker), and real exports (only available for AUT, GBR, and HUN). The key explanatory variable is the interaction term $\hbox {Crisis}_{ct} \times \hbox {EFD}_{cj}$ between the EFD measure (either from Compustat or EFIGE) and the dummy variable $\hbox {Crisis}_{ct}$, which equals one in the years of the banking crisis, as classified by the Worldbank’s Global Financial Development Database (GFDD, Cihak et al. 2012).^10^ In theory, we would expect that a negative credit supply shock in the crisis tightens existing credit constraints and thereby reduces the quantities of inputs employed and output produced by constrained firms (captured by $Y_{it}$).^11^ Furthermore, the effect of credit constraints should be stronger in industries that depend more on external finance, as shown theoretically by Manova (2013) for exports. Based on this hypothesis, we expect $\beta <0$.

Importantly, the firm fixed effect $\delta _{i}$ in Eq. (1) accounts for any time-invariant characteristics of firms, including size and productivity, country *c*, and industry affiliation *j*; therefore, it absorbs also the average effect of $\hbox {EFD}_{cj}$. The country-year fixed effect $\delta _{ct}$ controls for the overall crisis impact in each country and any other macroeconomic shocks. Eq. (1) is essentially a firm-level variant of the main specification by Dell’Ariccia et al. (2008), who assess the effects of banking crises on real performance in a panel of countries and industries. Compared to their specification, our approach cannot include industry-year fixed effects, because we look at a single crisis, but it has the significant advantage of exploiting within-firm variation.

### Main estimation results

Table 2 summarizes our results of estimating Eq. (1) for different performance variables and the two alternative EFD measures. When measuring EFD based on the EFIGE survey, our hypothesis is confirmed: all dimensions of firm performance were more negatively affected by the crisis in financially dependent industries compared to industries with low EFD. The estimated interaction effect is always negative and significant at conventional levels (with *p*-values in the range of 1–9%).

By contrast, the estimated interaction effect of the crisis with the Compustat index is zero for employment and exports, and we find a counter-intuitive positive interaction effect for turnover and labor productivity. These results indicate that *if* the credit crunch had a disproportionately negative impact on firm performance in high-EFD industries, in line with our hypothesis and the existing literature, then the industry-country-specific EFD measure from EFIGE is able to identify this effect for European firms, while the Compustat index, which relies on US data, is not.

**Table 2:** Differential crisis impact on firm performance by EFD

	Turnover		Employment		Labor productivity		Exports	
	(1)	(2)	(3)	(4)	(5)	(6)	(7)	(8)
Crisis $\times$EFD (EFIGE)	$-$ 0.075**		$-$ 0.041*		$-$ 0.059*		$-$ 0.083**	
	(0.036)		(0.024)		(0.032)		(0.032)	
Crisis $\times$ EFD (Compustat)		0.010**		0.001		0.005*		0.003
		(0.005)		(0.002)		(0.003)		(0.012)
Observations	707,039	707,039	505,612	505,612	396,084	396,084	91,791	91,791
Firms	190,418	190,418	167,537	167,537	124,434	124,434	27,177	27,177
Clusters	163	163	163	163	163	163	70	70
${R}^2$ (within firm)	0.114	0.114	0.013	0.013	0.056	0.056	0.029	0.029

The table shows OLS estimates of Eq. (1). The dependent variable for each column is the annual growth rate (in logs) of the respective variable indicated in the header. All regressions control for firm fixed effects and country-year fixed effects. Standard errors clustered by industry-country cell are reported in parentheses. Asterisks indicate significance levels: *$p<0.10$; **$\textit{p}<0.05$

### Shock transmission over time

We proceed by investigating the possibility that the credit supply shock propagated over time. It is conceivable that the credit crunch had delayed effects on firm performance, which our main specification in Eq. (1) admits only imperfectly. In particular, we have so far ignored the period after 2010, in which the crisis continued in some European countries. We now extend the analysis by using also data for the later years 2011–2013 and allow for delayed effects of the financial crisis on firm performance.

To achieve this, we estimate the following flexible version of our main econometric specification:

2 $$\begin{aligned} \Delta \ln Y_{it} = \sum _{\tau =2007}^{2013}\,{\tilde{\beta }}_\tau \,\textit{Y}_{\tau } \times \hbox {EFD}_{cj} + {\tilde{\delta }}_{ct} + {\tilde{\delta }}_{i} + {\tilde{\varepsilon }}_{it}, \end{aligned}$$

where we include a full set of interaction terms between $\hbox {EFD}_{cj}$ and year dummies $\textit{Y}_{\tau }$ for $\tau =\{2007,\dots ,2013\}$ (with 2006 as the base year). Estimates of the coefficients ${\tilde{\beta }}_\tau$ are informative about the timing of the differential crisis impact.

**Table 3:** Allowing for delayed effects of the crisis

	Turnover		Employment		Labor productivity		Exports	
	EFIGE	Compustat	EFIGE	Compustat	EFIGE	Compustat	EFIGE	Compustat
	(1)	(2)	(3)	(4)	(5)	(6)	(7)	(8)
$Y_{2007} \times$ EFD	$-$ 0.003	$-$ 0.009***	$-$ 0.015	$-$ 0.003	$-$ 0.032	$-$ 0.002	$-$ 0.007	$-$ 0.009
	(0.016)	(0.002)	(0.012)	(0.004)	(0.023)	(0.004)	(0.060)	(0.012)
$Y_{2008} \times$ EFD	$-$ 0.048	0.004	$-$ 0.026	$-$ 0.003	$-$ 0.062	0.006	$-$ 0.076	0.006
	(0.038)	(0.004)	(0.026)	(0.004)	(0.044)	(0.005)	(0.065)	(0.010)
$Y_{2009} \times$ EFD	$-$ 0.098*	0.013	$-$ 0.057*	0.004	$-$ 0.078	0.006	$-$ 0.165**	0.010
	(0.056)	(0.010)	(0.033)	(0.003)	(0.048)	(0.006)	(0.074)	(0.017)
$Y_{2010} \times$ EFD	$-$ 0.087**	$-$ 0.001	$-$ 0.056*	$-$ 0.002	$-$ 0.075**	$-$ 0.004	$-$ 0.038	$-$ 0.005
	(0.037)	(0.005)	(0.029)	(0.004)	(0.036)	(0.003)	(0.060)	(0.009)
$Y_{2011} \times$ EFD	$-$ 0.044	$-$ 0.009*	$-$ 0.043	$-$ 0.006*	$-$ 0.037	0.002	0.038	$-$ 0.023
	(0.036)	(0.005)	(0.032)	(0.004)	(0.029)	(0.004)	(0.083)	(0.017)
$Y_{2012} \times$ EFD	$-$ 0.031	$-$ 0.005	$-$ 0.045	$-$ 0.006**	$-$ 0.051	$-$ 0.008	$-$ 0.107*	$-$ 0.005
	(0.040)	(0.007)	(0.034)	(0.003)	(0.037)	(0.007)	(0.056)	(0.014)
$Y_{2013} \times$ EFD	$-$ 0.048	0.002	$-$ 0.064**	$-$ 0.007**	$-$ 0.033	0.006	$-$ 0.034	0.007
	(0.034)	(0.005)	(0.032)	(0.003)	(0.029)	(0.005)	(0.059)	(0.009)
Observations	1,168,899	1,168,899	886,490	886,490	667,332	667,332	144,475	144,475
${R}^2$	0.068	0.068	0.009	0.009	0.037	0.037	0.018	0.018

The table shows OLS estimates of Eq. (2). The dependent variable for each column is the annual growth rate (in logs) of the respective variable indicated in the header. All regressions control for firm fixed effects and country-year fixed effects. Standard errors clustered by industry-country cell are reported in parentheses. Asterisks indicate significance levels: $$*p<0.10$$; $$**p<0.05$$

Table 3 reports the estimation results. Two observations are worth noting. First, our key insights from the main analysis are confirmed: For each of the performance variables, the interaction effects of the EFD measure from the EFIGE survey are negative and significant for at least one of the crisis years 2009 and 2010. Concerning the timing, the point estimates suggest that the differential effect of the crisis on high vs. low EFD firms began to emerge in 2008, but became large and significant only in 2009 (or in 2010 for labor productivity). By contrast, none of the interaction effects is negative in 2009 for the EFD measure based on US Compustat data. Second, the evidence on delayed effects of the crisis is mixed. While the interaction effects of EFD (EFIGE) with the year dummies for 2011–2013 are all negative, they are rarely statistically significant (only for employment in 2013 and exports in 2012). Notably, the weak evidence on shock transmission over time may be due to the diverging economic development across European countries in those later years.

### Shock transmission across firms

It is well known that financial crises can trigger cascade effects, i.e., firms may be indirectly affected by the credit crunch via their business partners even if they are not credit constrained themselves. We now examine such spillovers across firms.

To this end, we add to Eq. (1) two interaction terms reflecting the EFD of firms’ customers and suppliers:

3 $$\begin{aligned} \Delta \ln Y_{it}= & {} {\check{\beta }} \,\hbox {Crisis}_{ct} \times \hbox {EFD}_{cj} + {\check{\gamma }} \,\hbox {Crisis}_{ct} \times \hbox {down EFD}_{cj} + {\check{\varphi }} \,\hbox {Crisis}_{ct} \times \hbox {up EFD}_{cj}\nonumber \\&+ \,{\check{\delta }}_{ct} + {\check{\delta }}_{i} + {\check{\varepsilon }}_{it}. \end{aligned}$$

We measure EFD for downstream firms (*down EFD*) using data on input-output linkages at the level of country-industry pairs for 2005 from the World Input Output Database (WIOD, Timmer et al. 2015). The index sums up the EFD measures across all country-industry pairs selling to country-industry *cj*, weighted by supply coefficients. This approach comes with two caveats: First, since our survey-based EFD measure is available only for seven countries, we impute for the remaining countries the industry-specific measure from the firm’s own country *j*. This choice is motivated by the fact that domestic suppliers and customers are of predominant importance for the majority of firms, so the domestic EFD measures seem to be the best available proxies. Second, EFD is measured only for manufacturing sectors, hence all non-manufacturing sectors receive a zero weight. EFD of upstream firms (*up EFD*) is computed analogously. If shock transmission via supply chains is relevant, we can expect to find negative estimates for the parameters ${\check{\gamma }}$ and ${\check{\varphi }}$.

**Table 4:** Shock transmission across firms via supply chains

	Turnover		Employment		Labor productivity		Exports	
	(1)	(2)	(3)	(4)	(5)	(6)	(7)	(8)
Crisis $$\times $$ EFD (EFIGE)	$$-$$ 0.031$$^{**}$$	$$-$$ 0.027$$^{*}$$	$$-$$ 0.017$$^{*}$$	$$-$$ 0.015	$$-$$ 0.024$$^{*}$$	$$-$$ 0.021	$$-$$ 0.033$$^{**}$$	$$-$$ 0.033$$^{**}$$
	(0.015)	(0.015)	(0.010)	(0.010)	(0.013)	(0.014)	(0.013)	(0.013)
Crisis $$\times $$ down EFD	$$-$$ 0.025$$^{***}$$	$$-$$ 0.007	$$-$$ 0.007$$^{***}$$	$$-$$ 0.003	$$-$$ 0.006	0.002	$$-$$ 0.008	$$-$$ 0.002
	(0.009)	(0.005)	(0.002)	(0.003)	(0.005)	(0.004)	(0.018)	(0.015)
Crisis $$\times $$ up EFD		$$-$$ 0.034$$^{***}$$		$$-$$ 0.009$$^{***}$$		$$-$$ 0.016$$^{***}$$		$$-$$ 0.016$$^{*}$$
		(0.005)		(0.003)		(0.004)		(0.009)
Observations	707,039	707,039	505,612	505,612	396,084	396,084	91,791	91,791
${R}^2$	0.114	0.115	0.013	0.013	0.056	0.056	0.029	0.029

The table shows OLS estimates of Eq. (3). The dependent variable for each column is the annual growth rate (in logs) of the respective variable indicated in the header. All regressions control for firm fixed effects and country-year fixed effects. Standard errors clustered by industry-country cell are reported in parentheses. Asterisks indicate significance levels: *$p<0.10$; **$p<0.05$, ***$p<0.01$

The estimation results reported in Table 4 strongly suggest that the propagation of the credit shock across firms matters indeed, notwithstanding the aforementioned measurement issues. We add the two interaction terms step by step: first the one involving *down EFD* and then also the one involving *up EFD*. For both variables, the estimates reveal disproportionate negative effects on firm performance in the crisis. While the differential effect of the crisis by downstream EFD is negative when added to the baseline specification, and significant for turnover and employment, it becomes insignificant in the combined regressions. Upstream EFD has a negative and significant interaction effect throughout, whether included individually (not reported) or in combined regressions. These estimates suggest that a high EFD of firms’ suppliers in the crisis significantly harmed their performance. Since all three EFD measures are standardized in these regressions (with mean zero and a standard deviation of one in the full estimation sample), we conclude that shock transmission across firms played a sizeable role in the financial crisis, comparable to the main differential effect of the crisis across sectors with different EFD.

### Robustness analysis

One might suspect that the estimations using the EFIGE measure suffer from an endogeneity issue due to reverse causality. If firms rated their industry lower in terms of EFD because they were hit harder by the crisis, this effect might bias our estimates of $\beta$ downward. Even though we cannot fully rule out such a bias, we have three reasons to believe that it is not driving our results.

First, the survey question is not concerned with the firm’s own current circumstances but targets general conditions in the industry. Second, for our results to be unbiased, we do not require that the reported EFD is entirely unaffected by the crisis. In particular, a uniform increase in the reported EFD of all firms in a given country would be absorbed by country-year fixed effects. Since the firms were surveyed simultaneously and since the crisis was highly synchronized across countries, as pointed out by Baldwin (2009) and confirmed in industry-level data,^12^ we would not expect the EFD ranking in 2010 to differ systematically from the fundamental ranking.

Third, in an important robustness check, we construct an alternative EFD measure based on questions in the EFIGE survey, which inquire about how the firm has financed its investments over the years 2007–2009. We compute the share of investments not financed internally for the median firm by industry, which directly reflects the idea of RZ. This alternative (also country-industry specific) EFD measure is based on the firm’s financial accounts and hence not prone to subjective judgment. The regressions using this measure, reported in Panel A of Table 5, generally confirm the differential crisis effects found in our main analysis, though the effects on employment and labor productivity are not statistically significant. These results further support the argument that the differences across EFD indexes documented in Table 1 are not merely driven by differences in measurement, but reflect inherent differences in EFD rankings across countries.

We conduct a series of additional robustness checks, which are detailed in Table 5. The pattern that we find in our main regressions is insensitive to (i) controlling for additional interaction terms of year dummies with industry characteristics (capital intensity, share of tangible assets, average firm size, and the Herfindahl index of turnover in 2005), (ii) including the years 2011–2013, coded as a non-crisis period, (iii) considering only the countries and industries for which the EFIGE EFD measure is based on at least ten firms, (iv) excluding potential outliers with extreme growth rates (top and bottom 1% of our dependent variables), and (v) excluding firms from the EFIGE pilot study when computing the EFIGE index. In these robustness checks, the interaction term of the crisis dummy with the EFIGE index of EFD is always estimated to be negative and remains statistically significant with few exceptions, while the interaction effect with the Compustat index is never negative and significant, in line with our main estimation results.

**Table 5:** Estimated interaction effects in robustness checks

	Turnover		Employment		Labor productivity		Exports	
	EFIGE	Compustat	EFIGE	Compustat	EFIGE	Compustat	EFIGE	Compustat
	(1)	(2)	(3)	(4)	(5)	(6)	(7)	(8)
*Panel A. Measuring EFD by the median share of investments not financed internally (from EFIGE)*								
*Crisis *$\times$ EFD	$-$ 0.045**		$-$ 0.010		$-$ 0.022		$-$ 0.042*	
	(0.021)		(0.008)		(0.014)		(0.024)	
Observations	707,039		505,612		396,084		91,791	
${R}^2$	0.114		0.013		0.056		0.029	
*Panel B. Controlling for interaction terms of year dummies with industry characteristics*								
*Crisis *$\times$ EFD	$-$ 0.075**	0.011**	$-$ 0.046*	0.000	$-$ 0.054*	0.006	$-$ 0.058	$-$ 0.006
	(0.037)	(0.005)	(0.024)	(0.003)	(0.029)	(0.003)	(0.035)	(0.010)
Observations	707,039	707,039	505,612	505,612	396,084	396,084	91,791	91,791
${R}^2$	0.115	0.115	0.013	0.013	0.057	0.057	0.030	0.030
*Panel C. Including 2011–2013 as a non-crisis period*								
*Crisis *$\times$ EFD	$-$ 0.052**	0.010**	$-$ 0.011	0.005***	$-$ 0.043**	0.003	$-$ 0.070***	0.009
	(0.020)	(0.004)	(0.009)	(0.002)	(0.019)	(0.002)	(0.025)	(0.009)
Observations	1,168,899	1,168,899	886,490	886,490	667,332	667,332	144,475	144,475
${R}^2$	0.068	0.068	0.009	0.009	0.037	0.037	0.018	0.018
*Panel D. Excluding small countries and industries*								
*Crisis *$\times$ EFD	$-$ 0.087*	0.011**	$-$ 0.046*	0.001	$-$ 0.060*	0.006**	$-$ 0.109**	0.003
	(0.047)	(0.005)	(0.026)	(0.002)	(0.034)	(0.003)	(0.046)	(0.013)
Observations	667,582	667,582	482,072	482,072	386,232	386,232	81,120	81,120
${R}^2$	0.118	0.118	0.013	0.013	0.057	0.057	0.026	0.026
*Panel E. Excluding potential outliers*								
*Crisis *$\times$ EFD	$-$ 0.066**	0.009**	$-$ 0.029	0.001	$-$ 0.047*	0.005**	$-$ 0.078**	0.003
	(0.031)	(0.004)	(0.020)	(0.002)	(0.028)	(0.002)	(0.032)	(0.007)
Observations	692,636	692,636	494,696	494,696	388,157	388,157	89,937	89,937
${R}^2$	0.230	0.230	0.026	0.025	0.098	0.098	0.043	0.043
*Panel F. Excluding EFIGE firms from the pilot study*								
*Crisis *$\times$ EFD	$-$ 0.061*		$-$ 0.037*		$-$ 0.044		$-$ 0.055**	
	(0.031)		(0.022)		(0.031)		(0.024)	
Observations	707,039		505,612		396,084		91,791	
${R}^2$	0.114		0.013		0.056		0.029	

The table shows the results of estimating (variations of) Eq. (1). The dependent variable for each column is the annual growth rate (in logs) of the respective variable indicated in the header. Odd columns report interaction effects for the EFIGE EFD measure, and even columns report interaction effects for the Compustat EFD measure. All regressions control for firm fixed effects and country-year fixed effects. In panel A, we measure EFD by one minus the industry median response to the question “How were these investments in plants, machines, equipment and ICT financed on average in the last three years (2007–2009)? Self-financing (use of internal sources)” (in percent/100) in EFIGE. In panel B, we add interaction terms of year dummies with the following industry characteristics: the logs of industry-level averages of capital intensity, the share of tangible assets, and operating revenues (as a proxy for firm size), as well as the Herfindahl index of operating revenues (all observed in 2005). In panel C, we extend the sample to include the years 2011–2013, in which we set the crisis dummy to zero. In panel D, we exclude the following small countries and industries for which the EFIGE EFD measure is in some instances based on less than ten firms: Austria, Hungary, “Tobacco” (NACE code 16), “Coke and refined petroleum products” (23), and “Office machinery and computers” (30). In panel E, we exclude potential outliers, defined as the observations with the highest and lowest 1% values of the respective dependent variable. In panel F, we exclude firms from the EFIGE pilot study when computing the EFIGE EFD measure. Standard errors clustered by country-industry cell are reported in parentheses. Asterisks indicate significance levels: *$p<0.10$; **$p<0.05$; ***$p<0.01$

## Concluding remarks

This paper has documented that industry rankings of a novel, survey-based index of external financial dependence (EFD) (i) are virtually uncorrelated with the standard index computed based on Compustat data for US firms following Rajan and Zingales (1998) and (ii) differ substantially across seven European countries. These findings suggest that an industry which is highly financially dependent in one country may rank low on EFD in another country. Investigating the fundamental determinants of these international differences is an exciting and promising area for future research.

Our results indicate that it might not be adequate to apply an EFD index computed from US data to other countries, as is currently standard practice in the literature. In a related paper, Ciccone and Papaioannou (2016) argue that this approach will cause a “benchmarking bias” if the US index is a less noisy proxy (i.e., a better benchmark) for some countries than for others. In light of these insights and our own results, we suggest that future research on financial dependence should not rely exclusively on the US index, but consider country-specific measures as complementary whenever possible.^13^ For European countries, the EFIGE index in Table 6 is a readily available option.

Finally, we have contributed to the literature investigating the real effects of the global financial crisis by providing comparable firm-level evidence across seven European countries. Our investigations using the survey-based EFD index suggest that the credit channel significantly contributed to reducing real firm performance in the crisis. We further contribute novel evidence pointing to the importance of input-output linkages in transmitting a credit shock: Firms’ crisis performance was significantly impaired if their customers and (in particular) their suppliers were highly financially dependent. These insights can guide policy makers in their efforts to preempt or mitigate the adverse effects of future financial crises.

## Figures and Tables

**Table 6:**
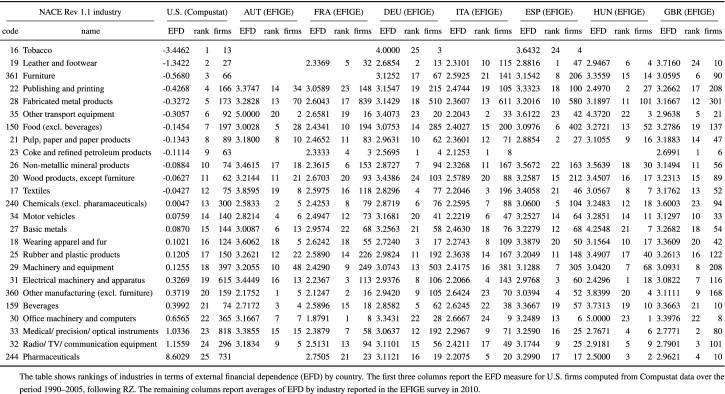
EFD rankings across countries

## A Data appendix

We closely follow RZ in calculating their index of external financial dependence (EFD). We use the North America Segment of the Compustat database to calculate the EFD measure over the time period 1990–2005.^14^ We only keep entries with cash flow statements (SCF) codes 1, 2, 3, and 7, and those with Industrial Format (INDFT $=$ INDL). Furthermore, we drop all firms for which the country of incorporation (FIC) is not the USA or for which the financial year (FYEAR) is missing.

As RZ, we construct EFD as the share of investment that cannot be financed through internal cash flows, i.e., capital expenditures minus cash flow from operations divided by capital expenditures, for the median firm by industry. Capital expenditures (CAPX) are readily available in Compustat. Following RZ, we define cash flow from operations as the sum of funds from operations and changes in working capital. For SCF codes 1, 2, and 3, we construct the cash-flow measure as the sum of total funds from operations (FOPT) plus increases in accounts payable ($\hbox {AP}_t -$ $\hbox {AP}_{t-1}$), decreases in inventories ($\hbox {INVT}_{t-1} -$ $\hbox {INVT}_{t}$), and decreases in receivables ($\hbox {RECT}_{t-1} -$ $\hbox {RECT}_{t}$) for each financial year *t*. For SCF code 7, total funds from operations are not available and therefore calculated as the sum of income before extraordinary items (IBC), depreciation and amortization (DPC), deferred taxes (TXDC), equity in net loss/earnings (ESUBC), sale of property, plant and equipment and investments (SPPIV), and other funds from operations (FOPO). We use the levels of the working capital variables and calculate the changes manually instead of using the reported changes provided by Compustat. This is because there are fewer missing values for the levels than for the changes in the dataset. Furthermore, we treat cash flow from operations as missing if any of its components is missing.

We calculate capital expenditures minus cash flow from operations for each firm over the period 1990–2005 and divide by the sum of capital expenditures over the respective time period, provided that both variables are non-missing. We then assign to each firm the 2-digit or 3-digit NACE Rev. 1.1 code (as reported in Table 6) corresponding to its SIC code.^15^ Finally, we use the median value by industry as our Compustat EFD index.

## Footnotes

1 While this conjecture is not the main subject of this paper, the global financial crisis of 2008, which originated in the USA, has revealed that US capital markets are still far from frictionless even in the twenty-first century.

2 At the time of this writing, the paper by RZ ranks among the top 100 most cited research papers in economics (https://ideas.repec.org/top) with more than 10,000 citations registered on Google scholar (https://scholar.google.com, both accessed on January 8, 2021).

3 The data were collected in the project “European Firms in a Global Economy” (Altomonte and Aquilante 2012, see http://bruegel.org/efige/).

4 In related work, von Furstenberg and von Kalckreuth (2006, 2007) use US data to investigate whether the Compustat index reflects fundamental industry characteristics.

5 The data contain representative samples for manufacturing firms with 10 or more employees in Austria (AUT), France (FRA), Germany (DEU), Hungary (HUN), Italy (ITA), Spain (ESP), and the UK (GBR).

6 See “Appendix A” for details of this procedure. In a related paper, Ferrando et al. (2008) compute the Compustat index for listed European firms.

7 Some firms in the EFIGE dataset were surveyed in a pilot study, some months before the main survey. Also, some industries host few firms. In unreported robustness checks, we confirm that the general picture of weak correlations in Table 1 remains unchanged after excluding firms from the pilot study or restricting the sample to countries and industries with at least ten observations.

8 Our seven-country sample does not offer sufficient cross-country variation in financial development to horse-race our new EFD measure against the original RZ specification. We therefore exploit the shock to credit conditions in the crisis for identification, which also offers the advantage of controlling for firm-specific effects.

9 Notice that the firm index *i* implies a unique country *c* and industry *j*, since information on locations and activities of firms in our data are static.

10 The GFDD indicates that the banking crisis started already in 2007 in GBR, but only in 2008 in the other six countries, and it did not end until 2010. Since the years after 2010 are not coded as a banking crisis in the GFDD, but still constitute a period of economic crisis in several European countries, we only use data for 2005–2010 in the main regressions. The full panel, including the years 2011–2013, is considered in further analyses in Sects. 4.3 and 4.5.

11 Empirical studies using linked firm-bank data have established a causal effect of credit supply shocks on firm employment (Chodorow-Reich 2013) and exports (Amiti and Weinstein 2011).

12 EFIGE questionnaires were completed by all firms (except those from the pilot study) between January and May 2010 (Altomonte and Aquilante 2012), while the majority of country-industry pairs in our sample experienced the steepest drop in monthly output between October 2008 and March 2009 (based on seasonally adjusted volume indices of production for 2-digit NACE industries from Eurostat).

13 Inklaar et al. (2015) also use an EFD measure based on balance sheets of European firms. Beck et al. (2005) use a firm survey measuring financial obstacles in more than 50 countries.
